# 3′-Hy­droxy­methyl-1′-methyl-3′-nitro-4′-(*o*-tol­yl)spiro­[indoline-3,2′-pyrrolidin]-2-one

**DOI:** 10.1107/S1600536810041917

**Published:** 2010-10-23

**Authors:** Rajeswari Gangadharan, K. SethuSankar, Manickam Bakthadoss, Nagappan Sivakumar, D. Velmurugan

**Affiliations:** a Department of Physics, Ethiraj College for Women (Autonomous), Chennai 600 008, India; b Department of Physics, R.K.M. Vivekananda College (Autonomous), Chennai 600 004, India; c Department of Organic Chemistry, University of Madras, Guindy Campus, Chennai 600 025, India; d Centre for Advanced Studies in Crystallography, University of Madras, Guindy Campus, Chennai 600 025, India

## Abstract

The title compound, C_20_H_21_N_3_O_4_, crystallizes with two mol­ecules in the asymmetric unit. In both mol­ecules, the pyrrolidine ring adopts an envelope conformation. The crystal structure is stabilized by inter­molecular C—H⋯O, N—H⋯O and O—H⋯O hydrogen bonds.

## Related literature

For ring puckering parameters, see: Cremer & Pople (1975 ▶).
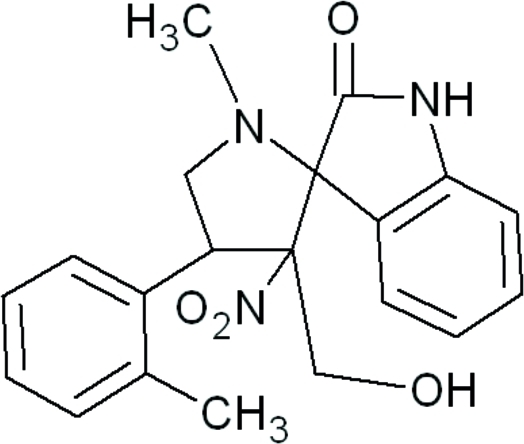


## Experimental

### 

#### Crystal data

C_20_H_21_N_3_O_4_
*M* *_r_* = 367.40
Triclinic, 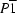

*a* = 10.8757 (18) Å
*b* = 13.625 (2) Å
*c* = 13.656 (2) Å
α = 66.116 (8)°
β = 83.715 (8)°
γ = 78.991 (9)°
*V* = 1814.9 (5) Å^3^
*Z* = 4
Mo *K*α radiation
μ = 0.10 mm^−1^
*T* = 293 K
0.30 × 0.26 × 0.20 mm

#### Data collection

Bruker APEXII CCD area detector diffractometer
32702 measured reflections
9209 independent reflections
5980 reflections with *I* > 2Σ(*I*)
*R*_int_ = 0.032

#### Refinement

*R*[*F* ^2^ > 2σ(*F* ^2^)] = 0.045
*wR*(*F* ^2^) = 0.131
*S* = 1.03
9209 reflections
493 parameters
H-atom parameters constrained
Δρ_max_ = 0.21 e Å^−3^
Δρ_min_ = −0.18 e Å^−3^

### 

Data collection: *APEX2* (Bruker, 2007 ▶); cell refinement: *SAINT* (Bruker, 2007 ▶); data reduction: *SAINT*; program(s) used to solve structure: *SHELXS97* (Sheldrick, 2008 ▶); program(s) used to refine structure: *SHELXL97* (Sheldrick, 2008 ▶); molecular graphics: *ORTEP-3* (Farrugia, 1997 ▶); software used to prepare material for publication: *SHELXL97* and *PLATON* (Spek, 2009 ▶).

## Supplementary Material

Crystal structure: contains datablocks global, I. DOI: 10.1107/S1600536810041917/bt5370sup1.cif

Structure factors: contains datablocks I. DOI: 10.1107/S1600536810041917/bt5370Isup2.hkl

Additional supplementary materials:  crystallographic information; 3D view; checkCIF report

## Figures and Tables

**Table 1:** Hydrogen-bond geometry (Å, °)

*D*—H⋯*A*	*D*—H	H⋯*A*	*D*⋯*A*	*D*—H⋯*A*
N3—H3*A*⋯O3^i^	0.86	2.00	2.782 (2)	151
O3—H3*B*⋯O7^ii^	0.82	2.11	2.7769 (17)	139
N5—H5⋯O8^iii^	0.86	2.39	3.0064 (19)	129
O7—H7⋯O4^iv^	0.82	2.24	2.9356 (19)	142
C8—H8⋯O1^v^	0.98	2.40	3.164 (2)	135
C33—H33*A*⋯O2^ii^	0.97	2.59	3.209 (2)	122

Symmetry codes: (i) 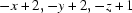; (ii) 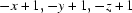; (iii) 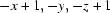; (iv) 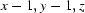; (v) 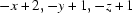.

**Fig. 1.**
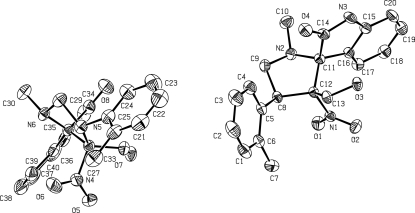
The molecular structure of title compound showing 50% probability displacement ellipsoids.

**Fig. 2.**
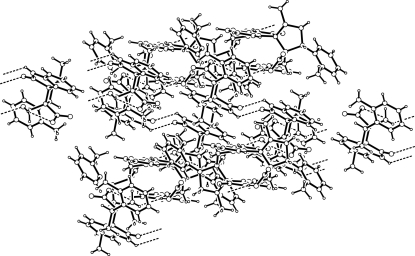
Packing diagram of the molecules viewed along the A axis.

## supplementary crystallographic information

### Comment

Substituted pyrrolidine compounds have gained much importance since they are the basic structural elements of many alkaloids and pharmacologically active compounds while molecules with the indole moiety posses anti-inflammatory and antibacterial properties In the title structure the asymmetric unit contains a pair of molecules with almost identical geometry. In the two molecules the bond lengths and angles agree with each other. In the benzene ring of the indole system the endocyclic angles at C17 and C20 are contracted to 118.78 (14)° and 117.64 (16)° respectively while those at C18, C19 and C15 are expanded to 120.92 (16)°, 121.19 (19)° and 121.56 (16)°, respectively. The sum of bond angles around N2 [331.33°] and  around  N3 atom [359.90°] indicate sp^3^ and sp^2^ hybridizations respectively. Dihedral angle formed between pyrrolidine ring and benzene ring is 71.98 (10)° and the dihedral angle between oxindole moiety and benzene ring is 45.04 (8)°. In addition to van der Waals interactions there are intermolecular C—H···O , N—H···O and O—H···O hydrogen bonds.

### Experimental

A mixture of (E) -2 nitro -3 *o*- tolyprop -2 en -1-ol(2 mmol,0.386 g) isatin (2 mmol,0.29 g) and sarcosine (2 mmol,0.18 g) in acetonitrile(8 mL) was refluxed for 5 h.After the completion of the reaction as indicated by TLC, the reaction mixture was concentrated and the resulting crude mass was diluted with water (10 ml) and extracted with ethyl acetate(3 *X* 10 ml).The combined organic layers were washed with brine and dried over anhydrous Na2 sO4. The organic layer was concentrated and the residue purified by column chromatography on Silica Gel (Acme 100–200 mesh),using ethyl acetate, hexanes (2:8) to afford final product as a olourless solid in 75% (0.55 g) yield.

### Refinement

H atoms were placed in idealized positions and allowed to ride on their parent atoms, with C—H bond lengths fixed to 0.93Å (aromatic H), 0.96Å (methyl H), 0.97Å (methylene H), 0.98Å (methine H), 0.82Å (OH), 0.86Å (NH), and *U*_iso_(H)=1.2 - 1.5 *U*_eq_(C,N).

### Crystal data

**Table:**

C_20_H_21_N_3_O_4_	*Z* = 4
*M**_r_* = 367.40	*F*(000) = 776
Triclinic, *P*1	*D*_x_ = 1.345 Mg m^−^^3^
Hall symbol: -P 1	Mo *K*α radiation, λ = 0.71073 Å
*a* = 10.8757 (18) Å	Cell parameters from 9209 reflections
*b* = 13.625 (2) Å	θ = 1.6–28.7°
*c* = 13.656 (2) Å	µ = 0.10 mm^−^^1^
α = 66.116 (8)°	*T* = 293 K
β = 83.715 (8)°	Black, black
γ = 78.991 (9)°	0.30 × 0.26 × 0.20 mm
*V* = 1814.9 (5) Å^3^	

### Data collection

**Table:**

Bruker APEXII CCD area detector diffractometer	5980 reflections with *I* > 2Σ(*I*)
Radiation source: fine-focus sealed tube	*R*_int_ = 0.032
graphite	θ_max_ = 28.7°, θ_min_ = 1.6°
ω and φ scans	*h* = −14→14
32702 measured reflections	*k* = −18→18
9209 independent reflections	*l* = −18→18

### Refinement

**Table:**

Refinement on *F*^2^	Primary atom site location: structure-invariant direct methods
Least-squares matrix: full	Secondary atom site location: difference Fourier map
*R*[*F*^2^ > 2σ(*F*^2^)] = 0.045	Hydrogen site location: inferred from neighbouring sites
*wR*(*F*^2^) = 0.131	H-atom parameters constrained
*S* = 1.03	*w* = 1/[σ^2^(*F*_o_^2^) + (0.0564*P*)^2^ + 0.3018*P*] where *P* = (*F*_o_^2^ + 2*F*_c_^2^)/3
9209 reflections	(Δ/σ)_max_ < 0.001
493 parameters	Δρ_max_ = 0.21 e Å^−^^3^
0 restraints	Δρ_min_ = −0.18 e Å^−^^3^

### Special details

**Table:**

Geometry. All e.s.d.'s (except the e.s.d. in the dihedral angle between two l.s. planes) are estimated using the full covariance matrix. The cell e.s.d.'s are taken into account individually in the estimation of e.s.d.'s in distances, angles and torsion angles; correlations between e.s.d.'s in cell parameters are only used when they are defined by crystal symmetry. An approximate (isotropic) treatment of cell e.s.d.'s is used for estimating e.s.d.'s involving l.s. planes.
Refinement. Refinement of *F*^2^ against ALL reflections. The weighted *R*-factor *wR* and goodness of fit *S* are based on *F*^2^, conventional *R*-factors *R* are based on *F*, with *F* set to zero for negative *F*^2^. The threshold expression of *F*^2^ > σ(*F*^2^) is used only for calculating *R*-factors(gt) *etc*. and is not relevant to the choice of reflections for refinement. *R*-factors based on *F*^2^ are statistically about twice as large as those based on *F*, and *R*- factors based on ALL data will be even larger.

### Fractional atomic coordinates and isotropic or equivalent isotropic displacement parameters (Å^2^)

**Table:**

	*x*	*y*	*z*	*U*_iso_*/*U*_eq_	
C1	0.9263 (2)	0.78067 (16)	0.12135 (15)	0.0643 (5)	
H1	0.8616	0.7728	0.0872	0.077*	
C2	1.0088 (2)	0.84968 (17)	0.06218 (15)	0.0736 (6)	
H2	1.0002	0.8872	−0.0113	0.088*	
C3	1.1032 (2)	0.86301 (17)	0.11143 (15)	0.0720 (6)	
H3	1.1588	0.9100	0.0715	0.086*	
C4	1.11632 (19)	0.80676 (14)	0.22033 (13)	0.0546 (4)	
H4	1.1808	0.8165	0.2533	0.066*	
C5	1.03477 (15)	0.73560 (12)	0.28191 (11)	0.0410 (3)	
C6	0.93751 (16)	0.72207 (13)	0.23161 (13)	0.0468 (4)	
C7	0.84219 (18)	0.65016 (16)	0.29240 (15)	0.0606 (5)	
H7A	0.8844	0.5780	0.3323	0.091*	
H7B	0.7877	0.6478	0.2429	0.091*	
H7C	0.7937	0.6788	0.3408	0.091*	
C8	1.04939 (14)	0.67486 (11)	0.40162 (11)	0.0366 (3)	
H8	1.0178	0.6063	0.4220	0.044*	
C9	1.18353 (15)	0.64548 (13)	0.43989 (12)	0.0444 (4)	
H9A	1.2255	0.5784	0.4341	0.053*	
H9B	1.2317	0.7031	0.3988	0.053*	
C10	1.28322 (17)	0.61668 (16)	0.60404 (15)	0.0585 (5)	
H10A	1.3231	0.6799	0.5690	0.088*	
H10B	1.3381	0.5542	0.5998	0.088*	
H10C	1.2650	0.6054	0.6779	0.088*	
C11	1.07282 (14)	0.72425 (10)	0.55415 (11)	0.0338 (3)	
C12	0.97488 (13)	0.73354 (10)	0.47288 (10)	0.0327 (3)	
C13	0.90941 (15)	0.84833 (11)	0.40830 (11)	0.0394 (3)	
H13A	0.9647	0.8835	0.3487	0.047*	
H13B	0.8349	0.8433	0.3787	0.047*	
C14	1.12455 (15)	0.83425 (12)	0.51371 (12)	0.0410 (3)	
C15	1.05989 (15)	0.79408 (12)	0.68790 (12)	0.0417 (3)	
C16	1.03173 (14)	0.70849 (11)	0.66827 (11)	0.0356 (3)	
C17	0.98993 (15)	0.62187 (12)	0.75116 (12)	0.0421 (3)	
H17	0.9736	0.5630	0.7395	0.051*	
C18	0.97263 (17)	0.62392 (14)	0.85264 (12)	0.0510 (4)	
H18	0.9442	0.5659	0.9093	0.061*	
C19	0.99680 (18)	0.71000 (16)	0.87043 (13)	0.0586 (5)	
H19	0.9835	0.7100	0.9389	0.070*	
C20	1.04078 (18)	0.79743 (16)	0.78823 (14)	0.0566 (5)	
H20	1.0568	0.8562	0.8003	0.068*	
N1	0.87235 (12)	0.66862 (9)	0.53968 (9)	0.0371 (3)	
N2	1.16682 (12)	0.63274 (9)	0.55149 (10)	0.0398 (3)	
N3	1.11411 (14)	0.86594 (11)	0.59629 (10)	0.0490 (3)	
H3A	1.1382	0.9239	0.5928	0.059*	
O1	0.89335 (12)	0.57011 (8)	0.57472 (9)	0.0532 (3)	
O2	0.77393 (11)	0.72006 (9)	0.55803 (10)	0.0551 (3)	
O3	0.87436 (11)	0.91441 (8)	0.46729 (8)	0.0444 (3)	
H3B	0.8182	0.8912	0.5112	0.067*	
O4	1.16951 (12)	0.88053 (9)	0.42497 (9)	0.0547 (3)	
C21	0.2916 (2)	0.53247 (16)	0.0557 (2)	0.0802 (7)	
H21	0.2397	0.5877	0.0049	0.096*	
C22	0.3238 (3)	0.54936 (18)	0.1412 (2)	0.0935 (8)	
H22	0.2922	0.6143	0.1490	0.112*	
C23	0.4028 (3)	0.46985 (19)	0.2153 (2)	0.0886 (7)	
H23	0.4263	0.4809	0.2731	0.106*	
C24	0.4473 (2)	0.37329 (16)	0.20326 (17)	0.0690 (5)	
H24	0.5022	0.3202	0.2528	0.083*	
C25	0.41187 (17)	0.35323 (13)	0.11889 (13)	0.0531 (4)	
C26	0.33357 (18)	0.43623 (15)	0.04218 (15)	0.0604 (5)	
C27	0.2939 (2)	0.42400 (18)	−0.05373 (17)	0.0790 (6)	
H27A	0.2390	0.4886	−0.0951	0.118*	
H27B	0.2509	0.3626	−0.0303	0.118*	
H27C	0.3666	0.4130	−0.0971	0.118*	
C28	0.45817 (16)	0.24525 (13)	0.10977 (12)	0.0486 (4)	
H28	0.4404	0.2556	0.0371	0.058*	
C29	0.59902 (17)	0.20421 (14)	0.12277 (14)	0.0554 (4)	
H29A	0.6458	0.2389	0.0567	0.066*	
H29B	0.6294	0.2175	0.1798	0.066*	
C30	0.73427 (17)	0.02658 (16)	0.17691 (16)	0.0635 (5)	
H30A	0.7592	0.0309	0.2402	0.095*	
H30B	0.7924	0.0560	0.1186	0.095*	
H30C	0.7337	−0.0481	0.1900	0.095*	
C31	0.51057 (15)	0.05040 (12)	0.23065 (11)	0.0413 (3)	
C32	0.39654 (15)	0.14565 (12)	0.18795 (12)	0.0418 (3)	
C33	0.30840 (17)	0.16622 (13)	0.27485 (13)	0.0484 (4)	
H33A	0.3405	0.2161	0.2962	0.058*	
H33B	0.2271	0.2017	0.2446	0.058*	
C34	0.54213 (17)	0.03808 (14)	0.34425 (12)	0.0503 (4)	
C35	0.52243 (17)	−0.13051 (14)	0.35819 (15)	0.0586 (5)	
C36	0.49706 (16)	−0.06504 (13)	0.25191 (14)	0.0501 (4)	
C37	0.47687 (19)	−0.11189 (17)	0.18355 (19)	0.0674 (5)	
H37	0.4634	−0.0696	0.1114	0.081*	
C38	0.4771 (2)	−0.2231 (2)	0.2245 (3)	0.0873 (8)	
H38	0.4622	−0.2553	0.1794	0.105*	
C39	0.4988 (2)	−0.28621 (19)	0.3300 (3)	0.0939 (9)	
H39	0.4970	−0.3605	0.3558	0.113*	
C40	0.5232 (2)	−0.24172 (17)	0.3991 (2)	0.0799 (7)	
H40	0.5396	−0.2849	0.4706	0.096*	
N4	0.31886 (15)	0.10989 (13)	0.12409 (13)	0.0566 (4)	
N5	0.55111 (16)	−0.06837 (12)	0.40917 (11)	0.0628 (4)	
H5	0.5723	−0.0947	0.4749	0.075*	
N6	0.60896 (12)	0.08871 (11)	0.14952 (10)	0.0466 (3)	
O5	0.23358 (14)	0.06078 (13)	0.17280 (14)	0.0789 (4)	
O6	0.34711 (16)	0.12759 (14)	0.03015 (11)	0.0842 (5)	
O7	0.29185 (12)	0.07180 (9)	0.36754 (9)	0.0552 (3)	
H7	0.2518	0.0347	0.3528	0.083*	
O8	0.55874 (14)	0.11090 (11)	0.36834 (9)	0.0677 (4)	

### Atomic displacement parameters (Å^2^)

**Table:**

	*U*^11^	*U*^22^	*U*^33^	*U*^12^	*U*^13^	*U*^23^
C1	0.0829 (15)	0.0693 (12)	0.0493 (10)	−0.0064 (11)	−0.0125 (10)	−0.0323 (9)
C2	0.1127 (19)	0.0718 (13)	0.0344 (9)	−0.0196 (13)	−0.0010 (11)	−0.0173 (9)
C3	0.1015 (17)	0.0757 (13)	0.0402 (10)	−0.0334 (12)	0.0126 (10)	−0.0194 (9)
C4	0.0684 (12)	0.0590 (10)	0.0405 (9)	−0.0226 (9)	0.0074 (8)	−0.0205 (8)
C5	0.0517 (10)	0.0396 (8)	0.0358 (7)	−0.0073 (7)	0.0033 (7)	−0.0203 (6)
C6	0.0548 (10)	0.0475 (9)	0.0445 (9)	−0.0053 (7)	−0.0011 (8)	−0.0261 (7)
C7	0.0559 (12)	0.0696 (12)	0.0645 (11)	−0.0155 (9)	−0.0081 (9)	−0.0310 (10)
C8	0.0442 (9)	0.0317 (7)	0.0368 (7)	−0.0077 (6)	0.0025 (6)	−0.0167 (6)
C9	0.0444 (9)	0.0455 (8)	0.0456 (8)	−0.0049 (7)	0.0041 (7)	−0.0227 (7)
C10	0.0453 (10)	0.0712 (12)	0.0550 (10)	−0.0050 (9)	−0.0069 (8)	−0.0215 (9)
C11	0.0404 (8)	0.0293 (7)	0.0319 (7)	−0.0098 (6)	0.0008 (6)	−0.0110 (5)
C12	0.0395 (8)	0.0281 (6)	0.0312 (7)	−0.0087 (5)	0.0026 (6)	−0.0118 (5)
C13	0.0519 (9)	0.0301 (7)	0.0348 (7)	−0.0035 (6)	−0.0003 (7)	−0.0128 (6)
C14	0.0481 (9)	0.0360 (7)	0.0407 (8)	−0.0160 (7)	−0.0003 (7)	−0.0134 (6)
C15	0.0458 (9)	0.0435 (8)	0.0396 (8)	−0.0128 (7)	−0.0010 (7)	−0.0179 (7)
C16	0.0392 (8)	0.0348 (7)	0.0328 (7)	−0.0072 (6)	−0.0019 (6)	−0.0126 (6)
C17	0.0459 (9)	0.0392 (8)	0.0369 (8)	−0.0083 (7)	−0.0016 (7)	−0.0099 (6)
C18	0.0516 (10)	0.0591 (10)	0.0323 (8)	−0.0101 (8)	0.0005 (7)	−0.0080 (7)
C19	0.0610 (12)	0.0828 (13)	0.0364 (9)	−0.0137 (10)	0.0013 (8)	−0.0277 (9)
C20	0.0658 (12)	0.0698 (11)	0.0518 (10)	−0.0194 (9)	−0.0012 (9)	−0.0383 (9)
N1	0.0420 (7)	0.0350 (6)	0.0373 (6)	−0.0106 (5)	0.0003 (5)	−0.0159 (5)
N2	0.0396 (7)	0.0377 (6)	0.0397 (7)	−0.0034 (5)	−0.0016 (5)	−0.0140 (5)
N3	0.0667 (10)	0.0435 (7)	0.0478 (8)	−0.0263 (7)	0.0024 (7)	−0.0227 (6)
O1	0.0643 (8)	0.0329 (6)	0.0619 (7)	−0.0176 (5)	0.0112 (6)	−0.0172 (5)
O2	0.0440 (7)	0.0520 (7)	0.0673 (8)	−0.0083 (5)	0.0129 (6)	−0.0249 (6)
O3	0.0569 (7)	0.0308 (5)	0.0453 (6)	−0.0079 (5)	0.0104 (5)	−0.0175 (5)
O4	0.0711 (9)	0.0487 (6)	0.0452 (6)	−0.0303 (6)	0.0112 (6)	−0.0137 (5)
C21	0.0712 (15)	0.0457 (11)	0.1031 (18)	−0.0113 (10)	−0.0016 (13)	−0.0078 (11)
C22	0.099 (2)	0.0495 (12)	0.128 (2)	−0.0200 (12)	0.0152 (17)	−0.0327 (14)
C23	0.117 (2)	0.0635 (14)	0.0929 (17)	−0.0343 (14)	0.0075 (16)	−0.0333 (13)
C24	0.0852 (15)	0.0542 (11)	0.0640 (12)	−0.0193 (10)	−0.0023 (11)	−0.0161 (9)
C25	0.0561 (11)	0.0463 (9)	0.0447 (9)	−0.0151 (8)	0.0064 (8)	−0.0046 (7)
C26	0.0514 (11)	0.0495 (10)	0.0607 (11)	−0.0142 (8)	0.0055 (9)	−0.0011 (8)
C27	0.0695 (14)	0.0789 (14)	0.0615 (12)	0.0009 (11)	−0.0153 (11)	−0.0025 (11)
C28	0.0518 (10)	0.0515 (9)	0.0320 (8)	−0.0074 (7)	−0.0005 (7)	−0.0064 (7)
C29	0.0496 (11)	0.0566 (10)	0.0442 (9)	−0.0135 (8)	0.0062 (8)	−0.0036 (8)
C30	0.0425 (10)	0.0709 (12)	0.0600 (11)	−0.0038 (9)	0.0063 (9)	−0.0128 (9)
C31	0.0396 (9)	0.0482 (8)	0.0321 (7)	−0.0079 (7)	0.0036 (6)	−0.0126 (6)
C32	0.0416 (9)	0.0473 (8)	0.0363 (8)	−0.0065 (7)	−0.0003 (7)	−0.0168 (7)
C33	0.0497 (10)	0.0459 (9)	0.0478 (9)	−0.0067 (7)	0.0086 (8)	−0.0195 (7)
C34	0.0542 (11)	0.0562 (10)	0.0318 (8)	−0.0052 (8)	0.0016 (7)	−0.0110 (7)
C35	0.0490 (11)	0.0504 (10)	0.0614 (11)	−0.0055 (8)	0.0202 (9)	−0.0134 (9)
C36	0.0391 (9)	0.0504 (9)	0.0562 (10)	−0.0062 (7)	0.0120 (8)	−0.0201 (8)
C37	0.0537 (12)	0.0693 (12)	0.0887 (15)	−0.0069 (9)	0.0082 (10)	−0.0448 (11)
C38	0.0610 (14)	0.0717 (15)	0.149 (3)	−0.0112 (11)	0.0154 (15)	−0.0679 (17)
C39	0.0608 (15)	0.0543 (13)	0.159 (3)	−0.0139 (11)	0.0330 (17)	−0.0414 (17)
C40	0.0603 (13)	0.0513 (11)	0.0947 (16)	−0.0041 (9)	0.0278 (12)	−0.0044 (11)
N4	0.0489 (9)	0.0652 (9)	0.0618 (10)	−0.0004 (7)	−0.0095 (8)	−0.0332 (8)
N5	0.0741 (11)	0.0574 (9)	0.0347 (7)	0.0013 (8)	0.0026 (7)	−0.0017 (7)
N6	0.0386 (8)	0.0545 (8)	0.0351 (7)	−0.0059 (6)	0.0063 (6)	−0.0086 (6)
O5	0.0528 (9)	0.0960 (11)	0.1116 (12)	−0.0257 (8)	0.0061 (8)	−0.0613 (10)
O6	0.0956 (12)	0.1114 (12)	0.0575 (9)	−0.0119 (9)	−0.0166 (8)	−0.0440 (8)
O7	0.0605 (8)	0.0497 (7)	0.0561 (7)	−0.0195 (6)	0.0224 (6)	−0.0230 (6)
O8	0.0923 (11)	0.0693 (8)	0.0421 (7)	−0.0152 (7)	−0.0121 (7)	−0.0194 (6)

### Geometric parameters (Å, °)

**Table:**

C1—C2	1.375 (3)	C21—C22	1.371 (4)
C1—C6	1.396 (2)	C21—C26	1.382 (3)
C1—H1	0.9300	C21—H21	0.9300
C2—C3	1.364 (3)	C22—C23	1.373 (4)
C2—H2	0.9300	C22—H22	0.9300
C3—C4	1.379 (2)	C23—C24	1.381 (3)
C3—H3	0.9300	C23—H23	0.9300
C4—C5	1.392 (2)	C24—C25	1.394 (3)
C4—H4	0.9300	C24—H24	0.9300
C5—C6	1.400 (2)	C25—C26	1.403 (3)
C5—C8	1.514 (2)	C25—C28	1.512 (2)
C6—C7	1.505 (3)	C26—C27	1.502 (3)
C7—H7A	0.9600	C27—H27A	0.9600
C7—H7B	0.9600	C27—H27B	0.9600
C7—H7C	0.9600	C27—H27C	0.9600
C8—C9	1.528 (2)	C28—C29	1.531 (2)
C8—C12	1.5660 (18)	C28—C32	1.565 (2)
C8—H8	0.9800	C28—H28	0.9800
C9—N2	1.4570 (19)	C29—N6	1.449 (2)
C9—H9A	0.9700	C29—H29A	0.9700
C9—H9B	0.9700	C29—H29B	0.9700
C10—N2	1.460 (2)	C30—N6	1.459 (2)
C10—H10A	0.9600	C30—H30A	0.9600
C10—H10B	0.9600	C30—H30B	0.9600
C10—H10C	0.9600	C30—H30C	0.9600
C11—N2	1.4638 (18)	C31—N6	1.460 (2)
C11—C16	1.5125 (19)	C31—C36	1.513 (2)
C11—C14	1.5657 (19)	C31—C34	1.561 (2)
C11—C12	1.5735 (19)	C31—C32	1.579 (2)
C12—C13	1.5251 (19)	C32—C33	1.524 (2)
C12—N1	1.5358 (18)	C32—N4	1.541 (2)
C13—O3	1.4121 (16)	C33—O7	1.4175 (19)
C13—H13A	0.9700	C33—H33A	0.9700
C13—H13B	0.9700	C33—H33B	0.9700
C14—O4	1.2141 (18)	C34—O8	1.213 (2)
C14—N3	1.3485 (19)	C34—N5	1.350 (2)
C15—C20	1.381 (2)	C35—N5	1.387 (3)
C15—C16	1.390 (2)	C35—C40	1.385 (3)
C15—N3	1.392 (2)	C35—C36	1.387 (3)
C16—C17	1.375 (2)	C36—C37	1.381 (3)
C17—C18	1.389 (2)	C37—C38	1.386 (3)
C17—H17	0.9300	C37—H37	0.9300
C18—C19	1.366 (3)	C38—C39	1.366 (4)
C18—H18	0.9300	C38—H38	0.9300
C19—C20	1.387 (3)	C39—C40	1.381 (4)
C19—H19	0.9300	C39—H39	0.9300
C20—H20	0.9300	C40—H40	0.9300
N1—O1	1.2121 (15)	N4—O6	1.221 (2)
N1—O2	1.2235 (16)	N4—O5	1.221 (2)
N3—H3A	0.8600	N5—H5	0.8600
O3—H3B	0.8200	O7—H7	0.8200
			
C2—C1—C6	121.46 (19)	C22—C21—C26	122.2 (2)
C2—C1—H1	119.3	C22—C21—H21	118.9
C6—C1—H1	119.3	C26—C21—H21	118.9
C3—C2—C1	119.93 (18)	C23—C22—C21	119.6 (2)
C3—C2—H2	120.0	C23—C22—H22	120.2
C1—C2—H2	120.0	C21—C22—H22	120.2
C2—C3—C4	120.0 (2)	C22—C23—C24	119.3 (2)
C2—C3—H3	120.0	C22—C23—H23	120.3
C4—C3—H3	120.0	C24—C23—H23	120.3
C3—C4—C5	121.21 (18)	C23—C24—C25	121.8 (2)
C3—C4—H4	119.4	C23—C24—H24	119.1
C5—C4—H4	119.4	C25—C24—H24	119.1
C4—C5—C6	118.91 (15)	C24—C25—C26	118.29 (18)
C4—C5—C8	120.78 (14)	C24—C25—C28	120.69 (16)
C6—C5—C8	120.31 (14)	C26—C25—C28	121.01 (17)
C1—C6—C5	118.50 (17)	C21—C26—C25	118.7 (2)
C1—C6—C7	118.91 (17)	C21—C26—C27	119.01 (19)
C5—C6—C7	122.55 (15)	C25—C26—C27	122.33 (18)
C6—C7—H7A	109.5	C26—C27—H27A	109.5
C6—C7—H7B	109.5	C26—C27—H27B	109.5
H7A—C7—H7B	109.5	H27A—C27—H27B	109.5
C6—C7—H7C	109.5	C26—C27—H27C	109.5
H7A—C7—H7C	109.5	H27A—C27—H27C	109.5
H7B—C7—H7C	109.5	H27B—C27—H27C	109.5
C5—C8—C9	115.73 (13)	C25—C28—C29	115.58 (15)
C5—C8—C12	115.67 (11)	C25—C28—C32	117.28 (13)
C9—C8—C12	104.24 (11)	C29—C28—C32	103.95 (12)
C5—C8—H8	106.9	C25—C28—H28	106.4
C9—C8—H8	106.9	C29—C28—H28	106.4
C12—C8—H8	106.9	C32—C28—H28	106.4
N2—C9—C8	103.39 (12)	N6—C29—C28	103.19 (14)
N2—C9—H9A	111.1	N6—C29—H29A	111.1
C8—C9—H9A	111.1	C28—C29—H29A	111.1
N2—C9—H9B	111.1	N6—C29—H29B	111.1
C8—C9—H9B	111.1	C28—C29—H29B	111.1
H9A—C9—H9B	109.0	H29A—C29—H29B	109.1
N2—C10—H10A	109.5	N6—C30—H30A	109.5
N2—C10—H10B	109.5	N6—C30—H30B	109.5
H10A—C10—H10B	109.5	H30A—C30—H30B	109.5
N2—C10—H10C	109.5	N6—C30—H30C	109.5
H10A—C10—H10C	109.5	H30A—C30—H30C	109.5
H10B—C10—H10C	109.5	H30B—C30—H30C	109.5
N2—C11—C16	110.87 (11)	N6—C31—C36	111.72 (13)
N2—C11—C14	113.72 (12)	N6—C31—C34	113.15 (13)
C16—C11—C14	101.40 (11)	C36—C31—C34	101.14 (13)
N2—C11—C12	102.28 (10)	N6—C31—C32	102.16 (12)
C16—C11—C12	120.25 (12)	C36—C31—C32	120.11 (14)
C14—C11—C12	108.79 (11)	C34—C31—C32	108.97 (12)
C13—C12—N1	106.79 (12)	C33—C32—N4	106.55 (13)
C13—C12—C8	113.48 (11)	C33—C32—C28	115.10 (13)
N1—C12—C8	109.38 (10)	N4—C32—C28	109.66 (12)
C13—C12—C11	115.71 (11)	C33—C32—C31	114.93 (12)
N1—C12—C11	106.58 (10)	N4—C32—C31	105.72 (12)
C8—C12—C11	104.63 (11)	C28—C32—C31	104.48 (13)
O3—C13—C12	114.57 (12)	O7—C33—C32	114.88 (13)
O3—C13—H13A	108.6	O7—C33—H33A	108.5
C12—C13—H13A	108.6	C32—C33—H33A	108.5
O3—C13—H13B	108.6	O7—C33—H33B	108.5
C12—C13—H13B	108.6	C32—C33—H33B	108.5
H13A—C13—H13B	107.6	H33A—C33—H33B	107.5
O4—C14—N3	126.40 (13)	O8—C34—N5	126.64 (16)
O4—C14—C11	125.96 (13)	O8—C34—C31	125.86 (15)
N3—C14—C11	107.60 (12)	N5—C34—C31	107.47 (15)
C20—C15—C16	121.56 (15)	N5—C35—C40	128.5 (2)
C20—C15—N3	128.19 (14)	N5—C35—C36	109.73 (15)
C16—C15—N3	110.14 (12)	C40—C35—C36	121.7 (2)
C17—C16—C15	119.84 (13)	C37—C36—C35	119.50 (17)
C17—C16—C11	130.98 (13)	C37—C36—C31	131.20 (17)
C15—C16—C11	108.58 (12)	C35—C36—C31	108.97 (15)
C16—C17—C18	118.78 (15)	C36—C37—C38	118.8 (2)
C16—C17—H17	120.6	C36—C37—H37	120.6
C18—C17—H17	120.6	C38—C37—H37	120.6
C19—C18—C17	120.92 (16)	C39—C38—C37	121.0 (2)
C19—C18—H18	119.5	C39—C38—H38	119.5
C17—C18—H18	119.5	C37—C38—H38	119.5
C18—C19—C20	121.19 (15)	C38—C39—C40	121.2 (2)
C18—C19—H19	119.4	C38—C39—H39	119.4
C20—C19—H19	119.4	C40—C39—H39	119.4
C15—C20—C19	117.64 (15)	C39—C40—C35	117.7 (2)
C15—C20—H20	121.2	C39—C40—H40	121.2
C19—C20—H20	121.2	C35—C40—H40	121.2
O1—N1—O2	123.13 (13)	O6—N4—O5	123.20 (17)
O1—N1—C12	119.39 (12)	O6—N4—C32	119.69 (16)
O2—N1—C12	117.43 (11)	O5—N4—C32	117.05 (15)
C9—N2—C10	114.40 (13)	C34—N5—C35	112.54 (15)
C9—N2—C11	106.64 (11)	C34—N5—H5	123.7
C10—N2—C11	115.29 (12)	C35—N5—H5	123.7
C14—N3—C15	112.25 (12)	C29—N6—C31	106.72 (12)
C14—N3—H3A	123.9	C29—N6—C30	114.73 (15)
C15—N3—H3A	123.9	C31—N6—C30	115.07 (12)
C13—O3—H3B	109.5	C33—O7—H7	109.5
			
C6—C1—C2—C3	−0.8 (3)	C26—C21—C22—C23	1.7 (4)
C1—C2—C3—C4	0.4 (3)	C21—C22—C23—C24	−1.0 (4)
C2—C3—C4—C5	0.2 (3)	C22—C23—C24—C25	−1.4 (4)
C3—C4—C5—C6	−0.4 (3)	C23—C24—C25—C26	3.0 (3)
C3—C4—C5—C8	−179.53 (16)	C23—C24—C25—C28	−177.8 (2)
C2—C1—C6—C5	0.6 (3)	C22—C21—C26—C25	0.0 (3)
C2—C1—C6—C7	178.36 (18)	C22—C21—C26—C27	179.9 (2)
C4—C5—C6—C1	0.0 (2)	C24—C25—C26—C21	−2.2 (3)
C8—C5—C6—C1	179.15 (14)	C28—C25—C26—C21	178.53 (17)
C4—C5—C6—C7	−177.67 (16)	C24—C25—C26—C27	177.82 (19)
C8—C5—C6—C7	1.5 (2)	C28—C25—C26—C27	−1.4 (3)
C4—C5—C8—C9	−31.83 (19)	C24—C25—C28—C29	−47.9 (2)
C6—C5—C8—C9	149.07 (14)	C26—C25—C28—C29	131.35 (17)
C4—C5—C8—C12	90.49 (18)	C24—C25—C28—C32	75.3 (2)
C6—C5—C8—C12	−88.62 (16)	C26—C25—C28—C32	−105.46 (18)
C5—C8—C9—N2	156.71 (11)	C25—C28—C29—N6	159.94 (13)
C12—C8—C9—N2	28.50 (14)	C32—C28—C29—N6	29.97 (16)
C5—C8—C12—C13	−5.69 (18)	C25—C28—C32—C33	−7.8 (2)
C9—C8—C12—C13	122.55 (13)	C29—C28—C32—C33	121.12 (15)
C5—C8—C12—N1	113.43 (13)	C25—C28—C32—N4	112.27 (16)
C9—C8—C12—N1	−118.33 (12)	C29—C28—C32—N4	−118.79 (14)
C5—C8—C12—C11	−132.72 (13)	C25—C28—C32—C31	−134.81 (14)
C9—C8—C12—C11	−4.48 (13)	C29—C28—C32—C31	−5.87 (16)
N2—C11—C12—C13	−146.49 (12)	N6—C31—C32—C33	−147.02 (13)
C16—C11—C12—C13	90.22 (15)	C36—C31—C32—C33	88.74 (17)
C14—C11—C12—C13	−25.90 (16)	C34—C31—C32—C33	−27.07 (18)
N2—C11—C12—N1	94.96 (11)	N6—C31—C32—N4	95.78 (14)
C16—C11—C12—N1	−28.33 (15)	C36—C31—C32—N4	−28.45 (18)
C14—C11—C12—N1	−144.44 (11)	C34—C31—C32—N4	−144.27 (13)
N2—C11—C12—C8	−20.86 (13)	N6—C31—C32—C28	−19.93 (14)
C16—C11—C12—C8	−144.14 (12)	C36—C31—C32—C28	−144.16 (14)
C14—C11—C12—C8	99.74 (12)	C34—C31—C32—C28	100.03 (14)
N1—C12—C13—O3	77.63 (15)	N4—C32—C33—O7	81.97 (17)
C8—C12—C13—O3	−161.78 (12)	C28—C32—C33—O7	−156.24 (14)
C11—C12—C13—O3	−40.80 (18)	C31—C32—C33—O7	−34.8 (2)
N2—C11—C14—O4	60.5 (2)	N6—C31—C34—O8	62.1 (2)
C16—C11—C14—O4	179.52 (16)	C36—C31—C34—O8	−178.23 (18)
C12—C11—C14—O4	−52.8 (2)	C32—C31—C34—O8	−50.8 (2)
N2—C11—C14—N3	−117.40 (14)	N6—C31—C34—N5	−116.05 (16)
C16—C11—C14—N3	1.64 (16)	C36—C31—C34—N5	3.58 (17)
C12—C11—C14—N3	129.34 (13)	C32—C31—C34—N5	131.05 (15)
C20—C15—C16—C17	−3.4 (2)	N5—C35—C36—C37	174.38 (16)
N3—C15—C16—C17	172.98 (14)	C40—C35—C36—C37	−2.6 (3)
C20—C15—C16—C11	−175.46 (15)	N5—C35—C36—C31	0.2 (2)
N3—C15—C16—C11	0.97 (18)	C40—C35—C36—C31	−176.80 (17)
N2—C11—C16—C17	−51.3 (2)	N6—C31—C36—C37	−54.8 (2)
C14—C11—C16—C17	−172.36 (16)	C34—C31—C36—C37	−175.49 (18)
C12—C11—C16—C17	67.8 (2)	C32—C31—C36—C37	64.7 (2)
N2—C11—C16—C15	119.53 (13)	N6—C31—C36—C35	118.41 (15)
C14—C11—C16—C15	−1.54 (15)	C34—C31—C36—C35	−2.24 (17)
C12—C11—C16—C15	−121.41 (14)	C32—C31—C36—C35	−122.05 (15)
C15—C16—C17—C18	2.2 (2)	C35—C36—C37—C38	2.8 (3)
C11—C16—C17—C18	172.19 (15)	C31—C36—C37—C38	175.49 (18)
C16—C17—C18—C19	−0.2 (3)	C36—C37—C38—C39	−1.0 (3)
C17—C18—C19—C20	−0.8 (3)	C37—C38—C39—C40	−1.1 (4)
C16—C15—C20—C19	2.4 (3)	C38—C39—C40—C35	1.3 (3)
N3—C15—C20—C19	−173.28 (17)	N5—C35—C40—C39	−175.85 (19)
C18—C19—C20—C15	−0.3 (3)	C36—C35—C40—C39	0.5 (3)
C13—C12—N1—O1	159.88 (12)	C33—C32—N4—O6	152.14 (16)
C8—C12—N1—O1	36.70 (16)	C28—C32—N4—O6	27.0 (2)
C11—C12—N1—O1	−75.88 (14)	C31—C32—N4—O6	−85.15 (18)
C13—C12—N1—O2	−22.68 (15)	C33—C32—N4—O5	−30.58 (19)
C8—C12—N1—O2	−145.86 (12)	C28—C32—N4—O5	−155.76 (15)
C11—C12—N1—O2	101.55 (14)	C31—C32—N4—O5	92.13 (17)
C8—C9—N2—C10	−173.13 (13)	O8—C34—N5—C35	178.02 (18)
C8—C9—N2—C11	−44.43 (14)	C31—C34—N5—C35	−3.8 (2)
C16—C11—N2—C9	170.06 (12)	C40—C35—N5—C34	179.13 (19)
C14—C11—N2—C9	−76.45 (14)	C36—C35—N5—C34	2.4 (2)
C12—C11—N2—C9	40.67 (14)	C28—C29—N6—C31	−45.54 (16)
C16—C11—N2—C10	−61.76 (16)	C28—C29—N6—C30	−174.26 (13)
C14—C11—N2—C10	51.73 (17)	C36—C31—N6—C29	170.46 (14)
C12—C11—N2—C10	168.84 (13)	C34—C31—N6—C29	−76.18 (16)
O4—C14—N3—C15	−179.06 (16)	C32—C31—N6—C29	40.80 (15)
C11—C14—N3—C15	−1.20 (18)	C36—C31—N6—C30	−61.02 (19)
C20—C15—N3—C14	176.30 (18)	C34—C31—N6—C30	52.34 (19)
C16—C15—N3—C14	0.18 (19)	C32—C31—N6—C30	169.33 (14)

### Hydrogen-bond geometry (Å, °)

**Table:**

*D*—H···*A*	*D*—H	H···*A*	*D*···*A*	*D*—H···*A*
N3—H3A···O3^i^	0.86	2.00	2.782 (2)	151
O3—H3B···O7^ii^	0.82	2.11	2.7769 (17)	139
N5—H5···O8^iii^	0.86	2.39	3.0064 (19)	129
O7—H7···O4^iv^	0.82	2.24	2.9356 (19)	142
C8—H8···O1^v^	0.98	2.40	3.164 (2)	135
C33—H33A···O2^ii^	0.97	2.59	3.209 (2)	122

Symmetry codes: (i) −*x*+2, −*y*+2, −*z*+1; (ii) −*x*+1, −*y*+1, −*z*+1; (iii) −*x*+1, −*y*, −*z*+1; (iv) *x*−1, *y*−1, *z*; (v) −*x*+2, −*y*+1, −*z*+1.
